# Inter-system reliability of markerless motion capture in older hip and knee patients during walking

**DOI:** 10.3389/fmedt.2026.1940851

**Published:** 2026-10-15

**Authors:** Leonhard Stein, Lukas Gschoßmann, Mareike Barthel, Sebastian Dendorfer

**Affiliations:** 1 Laboratory for Biomechanics, OTH Regensburg, Regensburg, Germany; 2 Research Center of Biomedical Engineering, University and OTH Regensburg, Regensburg, Germany; 3 Research Center of Health Sciences and Technology, University and OTH Regensburg, Regensburg, Germany

**Keywords:** gait analysis, inter-system, markerless, motion capture, older adults, osteoarthritis, reliability

## Abstract

This study evaluated the inter-system reliability of multi-camera markerless motion capture (MMC) during walking in older adults with hip or knee pathology and compared inter-system disagreement with a healthy control group. Forty-one participants were included: 15 hip patients, 13 knee patients, and 13 healthy controls. All participants performed walking trials recorded simultaneously by two temporally aligned MMC systems. Inter-system consistency was assessed using waveform-based comparison of joint angle trajectories across the gait cycle and discrete range of motion parameters. Waveform disagreement was quantified using one-dimensional statistical parametric mapping and root-mean-square error, while discrete outcomes were evaluated using intraclass correlation coefficient (ICC), standard error of measurement (SEM), minimal detectable change at the 95% confidence level, and Bland–Altman metrics. Group differences in subject-level inter-system disagreement metrics were tested using linear models. ICC-based inter-system reliability was predominantly high, with 18 of 36 parameters classified as excellent, 10 good, 6 moderate, and 2 poor. SEM values were below $2^{\circ }$ for most parameters. The least robust results were consistently observed for trunk flexion and selected transverse-plane variables. Exploratory group comparisons did not reveal a consistent directional pattern of increased disagreement in the pathological groups, and clinically plausible group differences were similarly reflected by both systems. However, the modest group sizes limit the ability to exclude smaller pathology-related effects on measurement consistency. These findings indicate small within-platform inter-system disagreement in most lower-limb outcomes for gait analysis in older adults with hip or knee pathology. Trunk and some transverse-plane outputs should be interpreted with greater caution.

## 1 Introduction

Three-dimensional motion analysis is widely used in biomechanics to quantify human movement in laboratory settings, but its routine clinical use remains limited. Marker-based motion capture, commonly regarded as the laboratory standard, requires substantial infrastructure, specialised personnel, and time-intensive preparation. Moreover, marker placement errors (1) and soft tissue artefacts (2) can distort estimated kinematics.

Markerless motion capture (MMC) systems may address several of these limitations by using computer vision and machine learning (ML) algorithms to estimate body segments and joint centres directly from video. This enables motion analysis in unprepared and fully clothed individuals and may improve feasibility in clinical settings (3).

Existing studies suggest that commercial multi-camera MMC systems like Theia3D (Theia Markerless, ON, CA) and Captury (DARI Motion, KS, US) can provide good validity for many kinematic outcomes, while also indicating that markerless and marker-based outputs should not be considered directly interchangeable (4–7).

However, established open-source and commercial pose-estimation algorithms are trained predominantly on typical humans in the wild (8) without indications of specific training on impaired individuals. Patients with hip or knee pathology frequently exhibit antalgic gait patterns that differ substantially from this training distribution (9, 10). Because markerless algorithms infer joint centres and segment orientations from visual priors rather than physical markers, atypical postures and compensatory strategies are plausible sources of degraded tracking performance, and domain shift has been shown to degrade pose-estimation accuracy when moving from healthy to pathological gait (11).

A related but distinct concern is the often reduced range of motion (RoM) in hip or knee patients (9, 10). Even if absolute measurement error is unchanged between groups, a smaller true RoM increases relative error and reduces the ability to detect a genuine post-operative change against measurement noise. Reliability benchmarks established in healthy or high-range-of-motion tasks may therefore be optimistic when applied to a population with compressed joint excursions, making population-specific error estimates clinically necessary rather than confirmatory.

Separately, ML models built on kinematic input are increasingly used to classify clinical gait patterns rather than to interpret individual joint angles (12). For this application, reliability may matter as much as validity, since unstable measurements directly reduce the reproducibility of downstream classification. Varcin and Boocock (13) therefore emphasised the need for rigorous reliability studies in pathological populations to clarify the clinical potential and limitations of ML-based tracking.

For Captury specifically, existing reliability studies have so far been restricted to healthy participants performing functional screening batteries, sports-related movements, or isolated single-joint test tasks (14–18). The absence of a study on clinical gait in older adults with pathological movement patterns constitutes a specific and clinically consequential gap, since objective, low-burden gait assessment could in principle be used to quantify functional impairment before hip or knee replacement and monitor recovery afterwards, reducing reliance on preparation-intensive marker-based testing. Realising this application requires that clinicians be able to distinguish a true post-operative change in gait from measurement noise. This distinction depends on population-specific estimates of measurement error and minimal detectable change, which is especially critical here given the compressed range of motion typical of this population. Inter-system disagreement under simultaneous acquisition represents one component of this error budget and provides a lower-bound estimate of measurement variability.

The present study addresses this gap by evaluating the inter-system reliability of Captury during walking in older patients with hip or knee pathology and by comparing inter-system disagreement with a healthy control group. Two Captury systems were used as parallel measurement units to assess inter-system consistency in both continuous waveform data and discrete RoM outcomes. The primary research question was: *How reliable is Captury for gait analysis in older adults with hip or knee pathology?* An exploratory secondary research question was: *What differences in inter-system disagreement are observed between the hip, knee, and healthy groups?*

## 2 Methods

### 2.1 Participants

A total of 41 participants (19 female, 22 male; age = 60.0 ± 17.6 years; BMI = 28.5 ± 5.1 kg/$m^{2}$; for details see Table 1) were divided into three groups: 15 hip patients (13x hip osteoarthritis, 1x hip dysplasia, 1x necrosis of the femoral head), 13 knee patients (knee osteoarthritis), and 13 healthy controls. Hip and knee patients presented with pain in the respective joints. Patient-reported joint-related health was assessed using the 12-item Hip disability and Osteoarthritis Outcome Score (HOOS-12) in the hip group and the 12-item Knee injury and Osteoarthritis Outcome Score (KOOS-12) in the knee group (19, 20). Both instruments provide pain, function, and quality of life scales and a summary score ranging from 0 to 100, with higher scores indicating better joint-related health. For healthy controls, both questionnaires were administered and the lower of the two summary scores was retained as a conservative indicator of lower-extremity joint-related health. Healthy participants were accompanying persons of the patients and were included if they reported no current lower-limb pain, no ongoing orthopaedic treatment, and no neurological or musculoskeletal disease affecting gait. Participants were recruited by convenience sampling from an orthopaedic clinic during the study period. Informed consent was obtained prior to data collection and ethical approval of the study according to the Declaration of Helsinki was obtained (permit number: 25-4347-101).

**Table 1:** Cohort characteristics.

Cohort	Sex		Age/Years		BMI/(kg/$m^{2}$)		Joint-related outcome score/100		Gait cycles/*n*	
	M	F	Mean	SD	Mean	SD	Mean	SD	Mean	SD
Control	7	6	47.5	22.0	26.0	4.7	93.5	13.0	26.9	4.3
Hip	10	5	64.1	13.4	29.1	3.7	53.9	17.1	29.9	7.5
Knee	5	8	67.8	8.9	30.2	6.2	52.7	14.3	34.0	8.0

For the hip and knee groups, joint-related outcome scores represent the HOOS-12 and KOOS-12 summary scores, respectively. For healthy controls, both questionnaires were administered and the lower summary score was retained. Scores range from 0 (worst) to 100 (best joint-related health). Gait-cycle counts represent the number of retained gait cycles per participant from the analysed limb.

### 2.2 Experimental set-up and procedure

Movements were recorded using two identical CapturyStudio systems. Each system comprised eight RGB machine-vision cameras (Blackfly S16S2C, Teledyne FLIR, OR, US) recording at 60 Hz with an auto-exposure setting below 15 ms and a resolution of 1,440×1,080p. The cameras of each system were arranged in a rectangular formation of approximately 10 m × 5 m around the 8 m walkway, including corner, anterior, posterior, and opposing lateral views (for camera setup schematic, see Supplementary Material). In each system, six cameras were wall-mounted and the two opposing lateral cameras were mounted on tripods, with camera heights ranging from 1.5 to 2.5 m. Corresponding cameras of the two systems were positioned approximately 0.2–0.3 m apart in three-dimensional space, resulting in small differences in horizontal position and height. Camera orientations were adjusted individually to provide comparable views directed towards the centre of the walkway. The two systems therefore represented practically equivalent, but not geometrically identical, camera configurations. Lighting and background conditions were kept constant. According to the manufacturer’s specifications, each system was calibrated separately, with a reported calibration residual below 1 mm.

Participants wore their own everyday indoor clothing in varying lengths and fits (trousers, T-shirts, and optional jumpers) as well as their own flat shoes.

All participants performed a dynamic scaling procedure to derive participant-specific segment dimensions for the Captury skeleton. Participants initially adopted a T-pose, standing upright with the arms abducted to approximately $90^{\circ }$, and subsequently moved the major upper- and lower-extremity joints through large ranges of motion. The sequence included ankle circumduction, hip and knee flexion, external hip rotation, forward trunk flexion with the legs extended, a deep squat, and corresponding movements of the shoulders, elbows, wrists, and head/neck. During this procedure, the software iteratively refined the participant-specific skeleton. Regardless of varying degrees of mobility restriction, all participants met the predefined internal criterion of ten different scaling poses. The final scaled skeleton was subsequently used for tracking the walking recordings; data from the scaling procedure itself were not included in the analysis.

Subsequently, participants completed eight consecutive passes along an 8 m walkway within one recording. Participants turned around after each traversal and continued with the subsequent pass. Walking speed was not externally controlled; participants were instructed to maintain their self-selected comfortable walking pace throughout the trial.

Synchronisation was carried out using an LED as a common event, and recordings were shifted to align with it. Subsequently, cross-correlation of knee flexion trajectories confirmed the absence of any residual temporal offset exceeding one frame.

### 2.3 Data processing

Raw video data from both systems were processed in CapturyStudio (v2.8.5) to extract 3D joint kinematics. Participant-specific segment lengths were derived from the scaling trials, and the resulting skeletons were used to track the gait recordings in the multisampling (4×) setting. According to the manufacturer, multisampling performs multiple tracking passes over a recorded sequence to identify the best tracking result.

Processing was performed on a workstation equipped with an AMD Ryzen 9 5900X 12-core CPU, 32 GB RAM, and an NVIDIA GeForce RTX 3090 GPU. On this configuration, initial tracking required approximately 3 min 20 s per minute of recorded sequence, and subsequent processing using the 4× multisampling setting required an additional approximately 3 min 24 s per recorded minute. Multisampling processing was performed in batches.

Gait cycles were segmented using an adapted kinematic heuristic (21), with cycle boundaries identified at the minimum vertical velocity of the ankle joint position between consecutive maxima of knee flexion. For each participant, a minimum of 15 gait cycles (see Table 1) from the analysed limb were retained from the central part of the walkway.

For the pathological groups, only the affected side was retained for lower-limb analysis. For healthy controls, the right side was selected a priori to retain one independent limb per participant. As a sensitivity analysis, all analyses were repeated using the left side of the healthy controls, which did not materially alter the interpretation of the results.

### 2.4 Statistical analysis

All statistical analyses were performed in Python 3.11.12 with a significance level of α=0.05. To address multiplicity, multiple comparisons correction was applied separately for each research question (22, 23).

#### 2.4.1 Inter-system reliability (research question 1)

Inter-system reliability and agreement between the two Captury systems were evaluated using discrete and waveform-based analyses.

##### 2.4.1.1 Discrete RoM parameters

For each joint and plane, RoM was defined within each gait cycle as the difference between the maximum and minimum joint angle (RoM=max−min). RoM was subsequently averaged across gait cycles within each participant and system before reliability analysis. Inter-system reliability was then quantified using a two-way random-effects, absolute-agreement, single-measure intraclass correlation coefficient (ICC(A,1)) with subject-level bootstrap-resampled confidence intervals. Intraclass correlation coefficient (ICC) values were classified as poor (<0.5), moderate (0.5–0.75), good (0.75–0.9), or excellent (>0.9) (24).

Additionally, standard error of measurement (SEM) (25), minimal detectable change at the 95% confidence level (${\text{MDC}}_{95}$) (26), %SEM (SEM/mean × 100), mean bias, and limits of agreement (LoA) (27) were calculated. SEM ≤ $2^{\circ }$ was considered acceptable for kinematics (28). Proportional bias was assessed by linearly regressing the inter-system difference on the mean of both systems (29).

All discrete reliability and agreement metrics were calculated separately for each group using subject-level means to avoid inflated precision from repeated gait cycles.

##### 2.4.1.2 Waveform comparison

Waveform differences across the normalised gait cycle (0%–100%) were assessed using one-dimensional statistical parametric mapping (SPM1D) (30). For each joint and plane, subject-level mean waveforms were computed per system, and paired t-tests were used to evaluate inter-system differences. To quantify disagreement magnitude, inter-system root-mean-square error (RMSE) was calculated from subject-level mean waveforms. Waveform analyses were conducted separately for each group. SPM1D results were interpreted together with RMSE magnitude.

#### 2.4.2 Group comparison of inter-system disagreement (research question 2)

In the secondary exploratory analysis, group differences in inter-system disagreement were evaluated using subject-level disagreement metrics. For discrete kinematics, disagreement was defined as the absolute difference in subject-level RoM between systems (|System 1 − System 2|). For waveform kinematics, disagreement was quantified as subject-level RMSE between systems across the normalised gait cycle. Group differences were tested for each joint–plane outcome using a linear model with group as a three-level categorical predictor. Pairwise contrasts were derived from the same model and expressed as the first minus the second group named in the contrast. Heteroscedasticity-consistent HC3 standard errors were used to account for possible variance inequalities, and 95% confidence intervals were calculated for all contrasts. Interpretation focused primarily on the estimated differences; non-significant tests were not interpreted as evidence of equivalence between groups. As a sensitivity analysis, model influence was assessed using standardised residuals, Cook’s distance, and leverage. Analyses were repeated after excluding the most influential participants identified across outcomes to evaluate result robustness.

## 3 Results

### 3.1 Waveform analysis

In the healthy control group (Figure 1), sagittal-plane kinematics showed no significant inter-system differences for the ankle, knee, or trunk, and only a brief difference for hip flexion during pre-swing. Within the sagittal plane, RMSE values exceeding $5^{\circ }$ occurred only for trunk flexion during swing. In the frontal plane, differences were briefly present for the ankle, hip and trunk, but corresponding RMSE values remained below $1.5^{\circ }$. Throughout most of the gait cycle, knee and hip rotation differed in the transverse plane. These differences partly remained significant after multiplicity correction, with peak RMSE values of $8.7^{\circ }$ and $9.8^{\circ }$, respectively.

**Figure 1:**
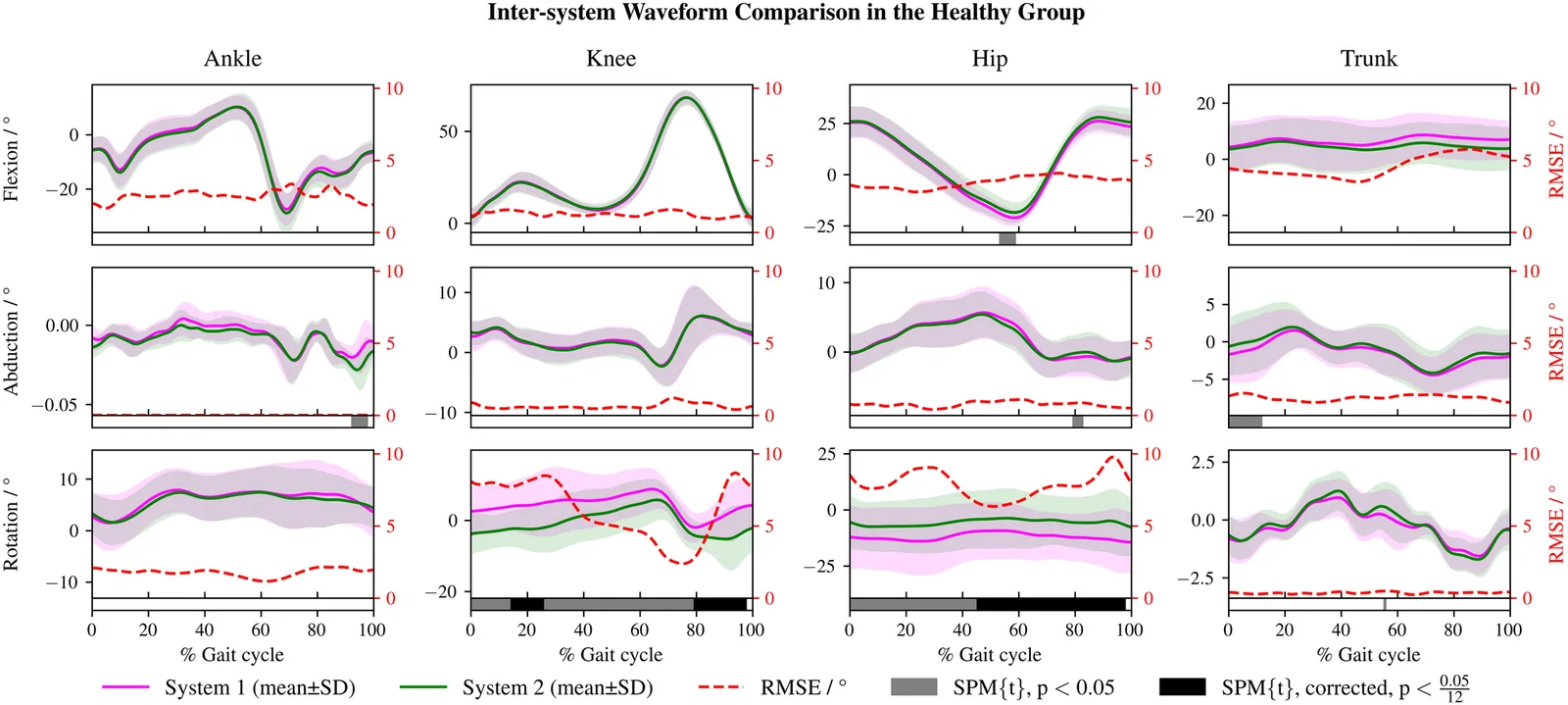
Inter-system waveform comparison in the healthy group. Mean ± standard deviation joint kinematics across the gait cycle (0%–100%) for the healthy group, comparing the two Captury systems. Columns represent joints (Ankle, Knee, Hip, Trunk), and rows represent movement planes (Flexion/Extension, Abduction/Adduction, Internal/External Rotation). For each panel, solid lines indicate the group mean waveform of System 1 (magenta) and System 2 (green); shaded areas represent ±1 standard deviation across subjects. The dashed red line (secondary y-axis) represents the root-mean-square error (RMSE) between systems, plotted on a common scale across all panels. SPM1D paired *t*-tests were used to assess inter-system differences across the gait cycle. The horizontal bar beneath each waveform indicates regions of significant difference. Grey and black segments denote regions significant at α=0.05 and significant after additional Bonferroni correction across the 12 joint–plane panels (α=0.05/12). All waveforms represent subject-level mean trajectories averaged across gait cycles prior to statistical analysis.

In the hip group (Figure 2), sagittal-plane differences were observed for knee, hip, and trunk kinematics, but lower-limb RMSE values remained below $5^{\circ }$. Trunk flexion showed the largest sagittal-plane disagreement, with RMSE values consistently above $5.5^{\circ }$ and peaking at $7.3^{\circ }$. Frontal-plane differences were observed briefly in the knee and scattered in hip kinematics, temporarily reaching corrected significance, with RMSE values below $1.7^{\circ }$. In the transverse plane, only a short difference was detected for trunk rotation. Lower-limb transverse-plane RMSE values were generally below $5^{\circ }$, with terminal-swing peaks for the knee and hip.

**Figure 2:**
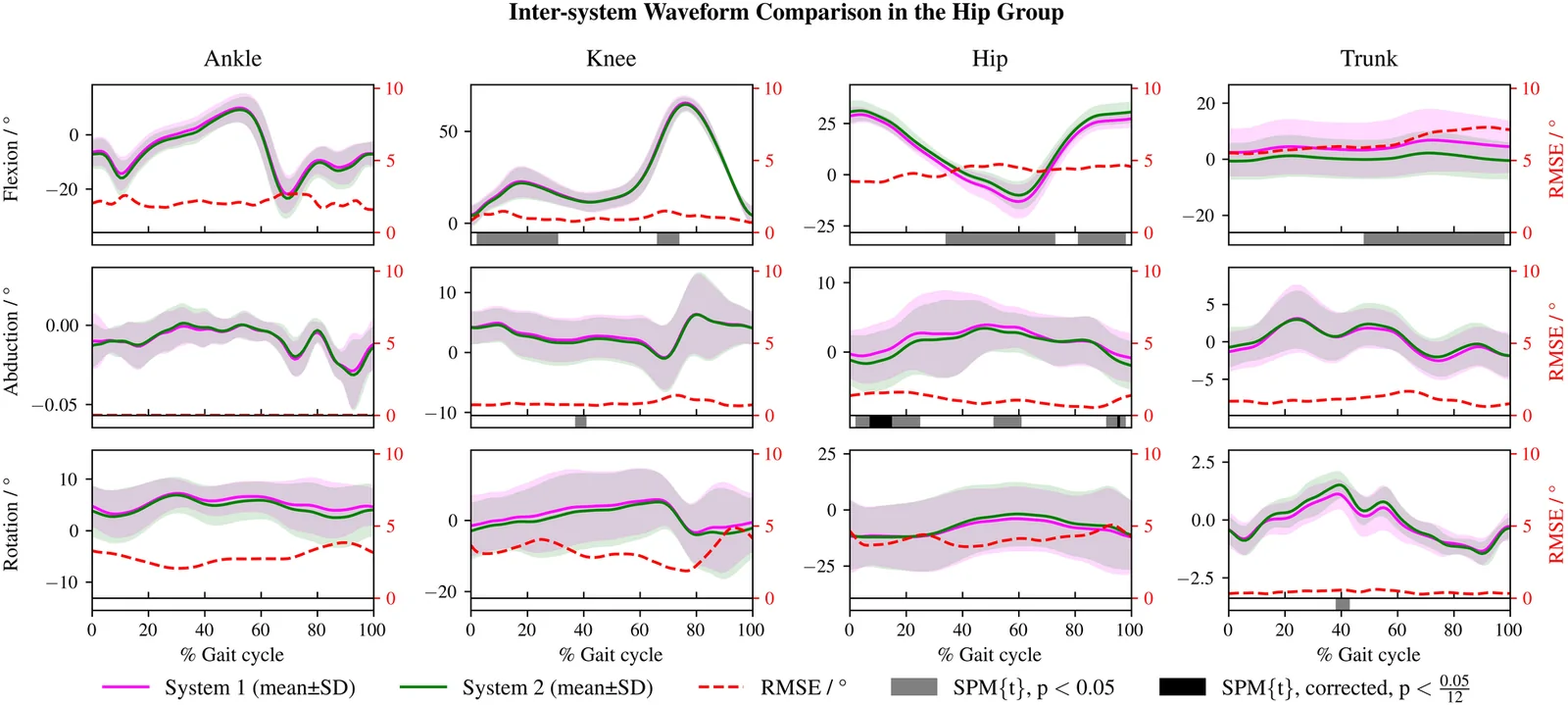
Inter-system waveform comparison in the hip group. Mean ± standard deviation joint kinematics across the gait cycle (0%–100%) for the hip group, comparing the two Captury systems. Columns represent joints (Ankle, Knee, Hip, Trunk), and rows represent movement planes (Flexion/Extension, Abduction/Adduction, Internal/External Rotation). For each panel, solid lines indicate the group mean waveform of System 1 (magenta) and System 2 (green); shaded areas represent ±1 standard deviation across subjects. The dashed red line (secondary y-axis) represents the root-mean-square error (RMSE) between systems, plotted on a common scale across all panels. SPM1D paired *t*-tests were used to assess inter-system differences across the gait cycle. The horizontal bar beneath each waveform indicates regions of significant difference. Grey and black segments denote regions significant at α=0.05 and significant after additional Bonferroni correction across the 12 joint–plane panels (α=0.05/12). All waveforms represent subject-level mean trajectories averaged across gait cycles prior to statistical analysis.

In the knee group (Figure 3), sagittal-plane differences were present for all lower-limb joints and for trunk flexion. Ankle and knee RMSE values remained below $5^{\circ }$, whereas hip flexion showed values between $4.0^{\circ }$ and $5.7^{\circ }$. Trunk flexion again showed the largest sagittal-plane disagreement, with RMSE values above $6.4^{\circ }$ and a peak of $8.1^{\circ }$. Frontal-plane differences were brief for the trunk and more extended and corrected significant during midstance for the hip, but RMSE values remained below $1.7^{\circ }$. In the transverse plane, differences were mainly observed for knee and hip rotation, with peak RMSE values of $6.4^{\circ }$ and $7.8^{\circ }$, respectively.

**Figure 3:**
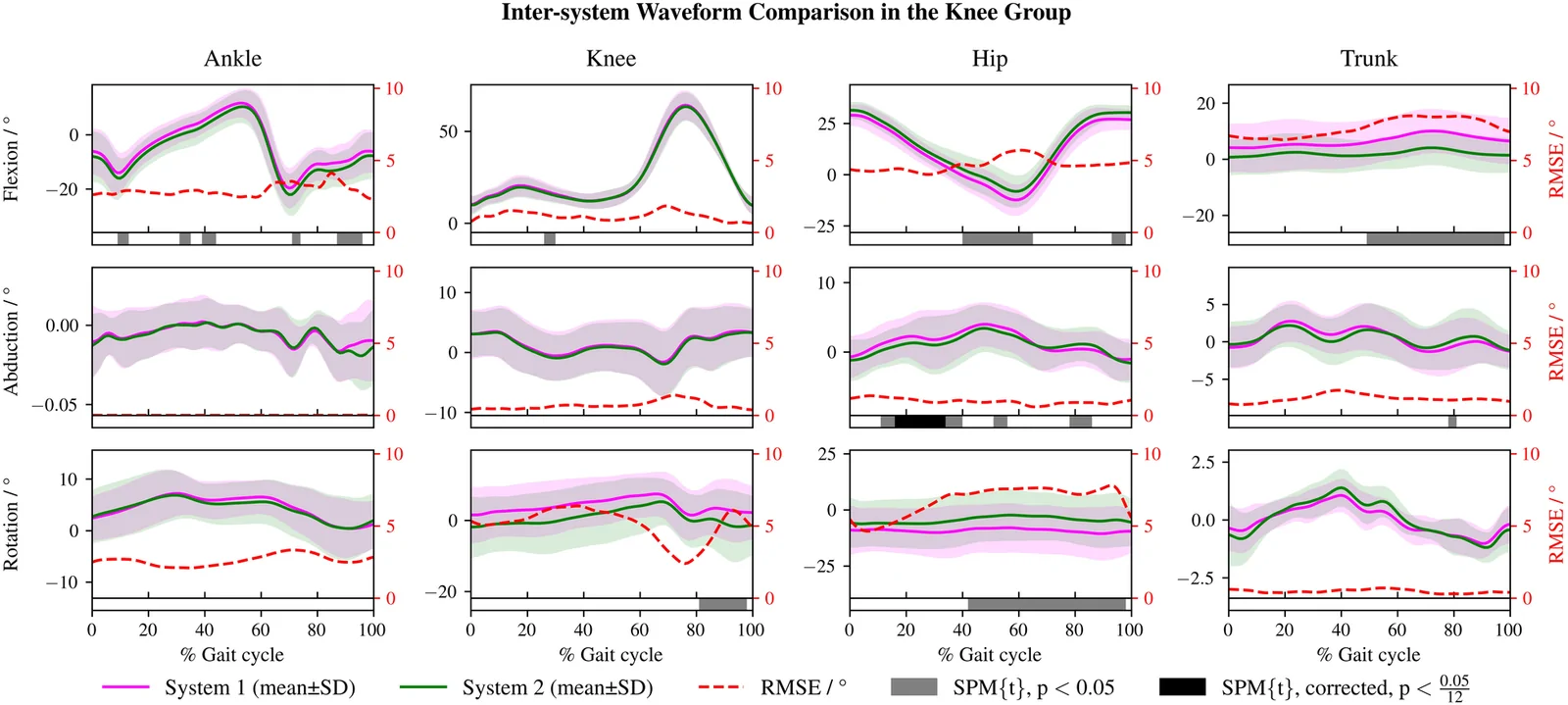
Inter-system waveform comparison in the knee group. Mean ± standard deviation joint kinematics across the gait cycle (0%–100%) for the knee group, comparing the two Captury systems. Columns represent joints (Ankle, Knee, Hip, Trunk), and rows represent movement planes (Flexion/Extension, Abduction/Adduction, Internal/External Rotation). For each panel, solid lines indicate the group mean waveform of System 1 (magenta) and System 2 (green); shaded areas represent ±1 standard deviation across subjects. The dashed red line (secondary y-axis) represents the root-mean-square error (RMSE) between systems, plotted on a common scale across all panels. SPM1D paired *t*-tests were used to assess inter-system differences across the gait cycle. The horizontal bar beneath each waveform indicates regions of significant difference. Grey and black segments denote regions significant at α=0.05 and significant after additional Bonferroni correction across the 12 joint–plane panels (α=0.05/12). All waveforms represent subject-level mean trajectories averaged across gait cycles prior to statistical analysis.

### 3.2 Discrete parameter analysis

The discrete RoM reliability results are summarised in Table 2. Across all analysed parameters (36 joint-plane-group combinations), ICC-based inter-system reliability was predominantly high, with 18 parameters classified as excellent, 10 good, 6 moderate, and 2 poor. Absolute measurement error was generally small. SEM was below $1^{\circ }$ for 20 parameters and between $1^{\circ }$ and $2^{\circ }$ for 12 parameters. Only 4 trunk flexion and hip rotation parameters exceeded $2^{\circ }$ SEM. Relative measurement error (%SEM) was low across most parameters. Knee flexion remained below 2% across all groups, while 10 parameters were between 2% and 5% and 10 between 5% and 10%. Errors above 10% occurred mainly in trunk and some rotational parameters. Systematic inter-system differences were minimal. In Bland–Altman analysis, 27 parameters showed a mean bias below $1^{\circ }$, and 6 parameters between $1^{\circ }$ and $2^{\circ }$. Larger biases ($2^{\circ }$–$4^{\circ }$) were observed only for trunk flexion across all groups. Proportional bias analysis revealed five parameters with significant relationships between inter-system difference and movement magnitude, indicating joint angle-dependent bias. No systematic pattern across joints, movement planes, or groups was found.

**Table 2:** Inter-system reliability of discrete range of motion parameters.

Joint	Plane	Group	ICC(A,1) [CI95]	Mean [SD]/${}^{\circ }$	SEM [MDC]/${}^{\circ }$	%SEM	${\mathrm{Bias}}_{\mathrm{abs}}$ [LoA]/${}^{\circ }$	${\mathrm{Bias}}_{\mathrm{prop}}$ (slope, *p*)
Trunk	Flx	Healthy	**0.69** [0.49, 0.82]	10.57 [4.17]	**2.18** [6.05]	**20.7**	**2.15** [−3.90, 8.21]	**0.69, p<0.001**
		Hip	**0.09** [−0.14, 0.43]	9.83 [1.20]	1.42 [3.94]	**14.5**	**2.11** [−1.84, 6.05]	0.76, p=0.090
		Knee	**0.42** [0.21, 0.84]	10.91 [3.82]	**3.15** [8.73]	**28.9**	**2.93** [−5.80, 11.66]	**0.91, p=0.002**
	Abd	Healthy	0.95 [0.89, 0.98]	10.49 [3.71]	0.84 [2.33]	8.0	−0.07 [−2.40, 2.27]	−0.07, p=0.506
		Hip	0.85 [0.67, 0.96]	10.28 [2.67]	1.10 [3.04]	**10.7**	0.02 [−3.02, 3.07]	0.17, p=0.288
		Knee	**0.58** [0.05, 0.87]	9.30 [1.81]	1.33 [3.67]	**14.2**	−0.41 [−4.09, 3.26]	0.15, p=0.643
	Rot	Healthy	0.96 [0.88, 0.98]	4.23 [0.99]	0.18 [0.51]	4.4	−0.15 [−0.66, 0.36]	−0.01, p=0.901
		Hip	0.77 [0.48, 0.91]	4.20 [0.68]	0.30 [0.84]	7.2	−0.28 [−1.12, 0.55]	0.10, p=0.582
		Knee	0.77 [0.64, 0.94]	4.13 [1.01]	0.44 [1.21]	**10.6**	−0.44 [−1.65, 0.77]	**−0.49, p=0.001**
Hip	Flx	Healthy	0.93 [0.69, 0.96]	49.18 [5.81]	1.38 [3.83]	2.8	1.21 [−2.62, 5.04]	0.10, p=0.312
		Hip	0.99 [0.97, 1.00]	44.91 [8.24]	0.64 [1.77]	1.4	0.57 [−1.21, 2.34]	−0.03, p=0.292
		Knee	0.92 [0.81, 0.97]	44.36 [7.57]	1.89 [5.24]	4.3	1.81 [−3.43, 7.05]	0.13, p=0.211
	Abd	Healthy	0.96 [0.78, 0.99]	10.59 [3.81]	0.71 [1.98]	6.7	−0.62 [−2.60, 1.36]	0.03, p=0.715
		Hip	0.92 [0.71, 0.97]	9.65 [2.47]	0.74 [2.04]	7.6	−0.27 [−2.30, 1.77]	0.15, p=0.179
		Knee	0.86 [0.53, 0.94]	9.30 [1.68]	0.66 [1.83]	7.1	−0.22 [−2.05, 1.61]	−0.14, p=0.393
	Rot	Healthy	**0.67** [0.17, 0.92]	15.22 [3.99]	**2.57** [7.12]	**16.9**	−0.30 [−7.42, 6.82]	−0.10, p=0.718
		Hip	0.84 [0.39, 0.94]	18.05 [5.70]	**2.31** [6.40]	**12.8**	−1.13 [−7.53, 5.27]	0.16, p=0.303
		Knee	0.89 [0.40, 0.97]	16.24 [4.13]	1.47 [4.06]	9.0	−0.18 [−4.25, 3.88]	0.15, p=0.330
Knee	Flx	Healthy	0.98 [0.94, 0.99]	67.39 [4.80]	0.67 [1.86]	1.0	0.32 [−1.54, 2.17]	−0.07, p=0.212
		Hip	0.99 [0.98, 1.00]	63.11 [6.00]	0.51 [1.40]	0.8	0.42 [−0.98, 1.82]	−0.03, p=0.411
		Knee	0.99 [0.87, 1.00]	58.01 [7.71]	0.60 [1.67]	1.0	0.47 [−1.20, 2.14]	0.01, p=0.829
	Abd	Healthy	0.98 [0.92, 0.99]	11.75 [3.46]	0.47 [1.31]	4.0	−0.41 [−1.72, 0.90]	−0.03, p=0.595
		Hip	0.98 [0.96, 0.99]	12.36 [3.40]	0.36 [1.00]	2.9	−0.36 [−1.36, 0.65]	**−0.08, p=0.049**
		Knee	0.95 [0.85, 0.99]	10.59 [1.95]	0.44 [1.23]	4.2	−0.04 [−1.27, 1.19]	−0.03, p=0.793
	Rot	Healthy	0.84 [0.62, 0.93]	18.86 [3.99]	1.69 [4.68]	9.0	−0.64 [−5.32, 4.05]	−0.10, p=0.572
		Hip	0.92 [0.80, 0.96]	19.71 [3.92]	1.17 [3.25]	5.9	−0.16 [−3.40, 3.09]	0.01, p=0.903
		Knee	0.88 [0.51, 0.96]	16.71 [2.34]	0.81 [2.26]	4.9	−0.46 [−2.71, 1.80]	−0.02, p=0.901
Ankle	Flx	Healthy	0.94 [0.79, 0.96]	41.55 [5.76]	0.85 [2.37]	2.1	−1.77 [−4.14, 0.60]	−0.07, p=0.244
		Hip	0.95 [0.86, 0.98]	36.06 [5.45]	0.84 [2.33]	2.3	−1.43 [−3.76, 0.90]	0.04, p=0.528
		Knee	0.98 [0.93, 0.99]	38.49 [7.97]	1.01 [2.81]	2.6	−1.14 [−3.95, 1.67]	−0.09, p=0.086
	Abd	Healthy	**0.72** [0.40, 0.90]	0.06 [0.01]	0.01 [0.02]	**11.3**	−0.00 [−0.02, 0.02]	−0.34, p=0.158
		Hip	0.86 [0.60, 0.97]	0.07 [0.01]	0.01 [0.01]	7.7	−0.00 [−0.01, 0.01]	0.14, p=0.380
		Knee	**0.67** [0.18, 0.92]	0.06 [0.01]	0.01 [0.02]	**10.6**	−0.00 [−0.02, 0.02]	0.13, p=0.655
	Rot	Healthy	0.79 [0.56, 0.88]	12.94 [3.88]	1.88 [5.22]	**14.6**	−0.59 [−5.82, 4.63]	**0.51, p=0.004**
		Hip	**0.68** [0.32, 0.87]	12.33 [2.30]	1.43 [3.97]	**11.6**	−0.43 [−4.40, 3.54]	0.21, p=0.396
		Knee	0.90 [0.74, 0.95]	12.89 [3.40]	1.15 [3.18]	8.9	0.21 [−2.97, 3.39]	0.18, p=0.215

RoM metrics are presented separately for each *Joint*, *Plane*, and *Group* (healthy control, hip, or knee group). RoM values were averaged at subject level prior to reliability analysis to ensure independence of observations. For each parameter, the table reports the mean across both systems, the standard deviation of the subject-level paired-system means (Mean [SD]/${}^{\circ }$), and the two-way random-effects, absolute-agreement, single-measure intraclass correlation coefficient (ICC(A,1) [95% CI]). Absolute measurement error is quantified by the standard error of measurement and minimal detectable change at the 95% confidence level (SEM [${\mathrm{MDC}}_{95}$]/${}^{\circ }$), and relative measurement error by %SEM. Systematic and random inter-system differences according to Bland–Altman analysis are described by mean bias with limits of agreement (Bias [LoA]/${}^{\circ }$) and by the proportional bias test (regression slope and p-value). To facilitate interpretation, values exceeding predefined thresholds are highlighted in bold: ICC < 0.75, SEM > $2^{\circ }$, %SEM > 10%, Bias > $2^{\circ }$, and proportional bias when p<0.05. Lower SEM, ${\mathrm{MDC}}_{95}$, and Bias values indicate smaller measurement error or systematic differences, whereas higher ICC values indicate better relative reliability.

### 3.3 Group comparison

Estimated group differences (Figure 4) varied in direction across parameters and did not reveal a consistent pattern of greater disagreement in either pathological group (detailed results in Supplementary Tables). None of the 72 contrasts remained significant after Benjamini–Hochberg false discovery rate (FDR) adjustment. Nominal group differences were observed for knee flexion in both discrete and waveform disagreement for the knee–hip comparison and in discrete disagreement for the healthy–knee comparison. The magnitude of the group differences did not exceed $0.5^{\circ }$ in knee flexion. Additional nominal contrasts were observed for ankle and trunk rotation as well as hip abduction, with differences of $1^{\circ }$, $0.3^{\circ }$ and $0.5^{\circ }$, respectively. The largest nominal differences ranged from $2^{\circ }$ to $3.5^{\circ }$ and were observed in trunk flexion as well as knee and hip rotation.

**Figure 4:**
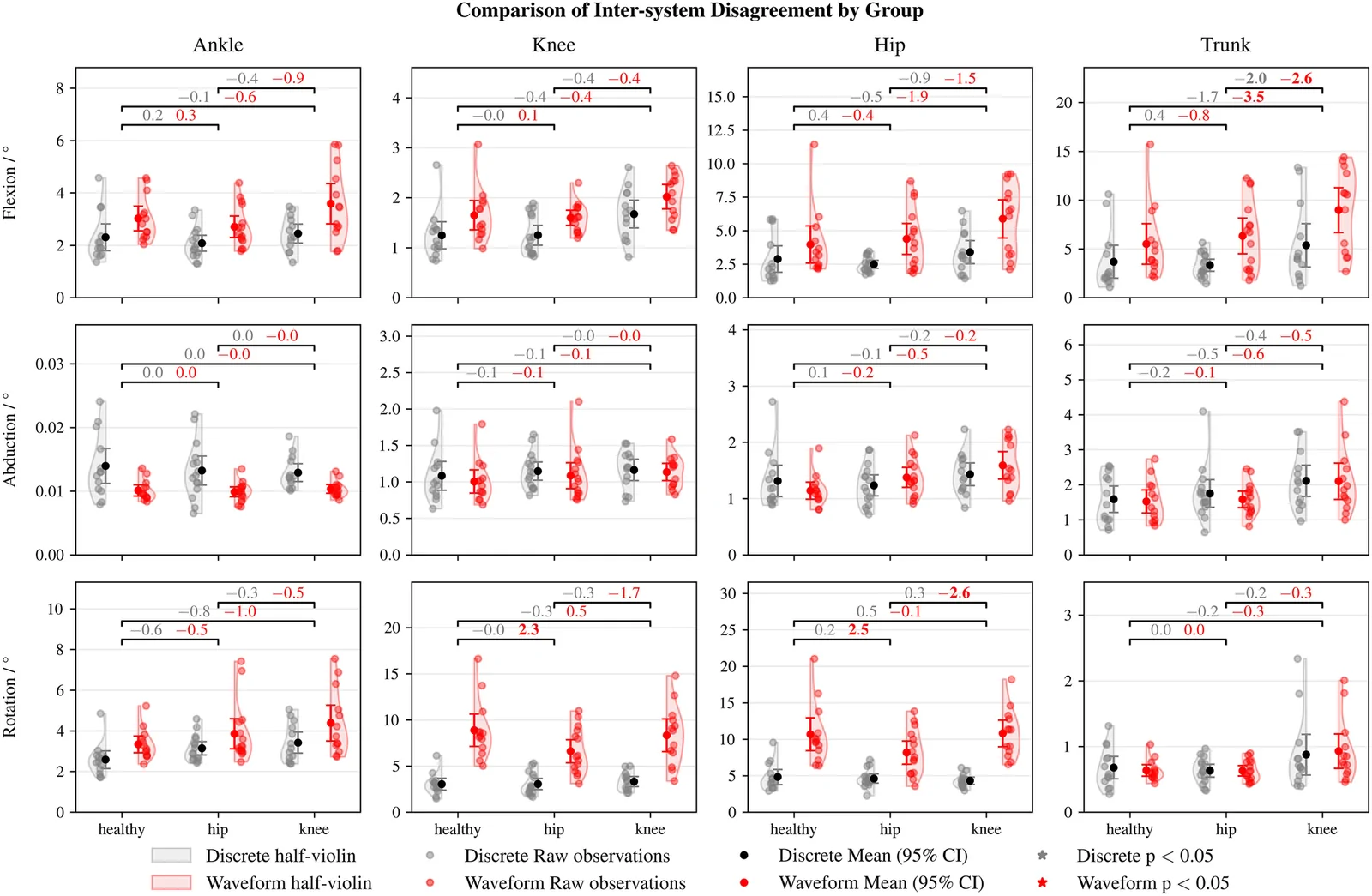
Subject-level group differences in inter-system disagreement for discrete range of motion (RoM) and waveform root-mean-square error (RMSE) across the normalised gait cycle. For each joint–plane combination, grey distributions represent the subject-level mean absolute between-system difference in RoM, and red distributions represent the subject-level waveform RMSE between systems. Groups shown are healthy controls, hip, and knee patients. Lines indicate pairwise group contrasts. Grey values represent discrete RoM disagreement, and red values represent waveform RMSE. Correspondingly coloured numbers above each contrast line show the model-estimated group difference, calculated as the first group minus the second group. Contrast estimates with an absolute magnitude exceeding $2^{\circ }$ are shown in bold. Grey and red stars indicate discrete and waveform contrasts, respectively, with a per-family Benjamini–Hochberg false discovery rate-adjusted p<0.05 (none present). Corresponding 95% confidence intervals are reported in Supplementary Tables.

## 4 Discussion

The present study evaluated the inter-system reliability of the MMC system Captury during walking in older adults with hip or knee pathology and in healthy controls. Using temporally aligned recordings from two independent systems, inter-system consistency was assessed with continuous waveform analyses and discrete RoM parameters.

Overall, the results showed high inter-system reliability for most lower-extremity kinematic outcomes. Discrete analyses indicated small measurement errors for the majority of parameters, and waveform analyses showed generally low disagreement magnitudes. No systematic pattern of greater inter-system disagreement was observed in participants with hip or knee pathology compared with healthy controls. However, the exploratory group comparisons were not sufficiently precise to exclude smaller pathology-related effects or to establish equivalent measurement consistency between groups. The least robust results were observed for trunk flexion and selected transverse-plane variables, which should therefore be interpreted more cautiously than sagittal-plane lower-extremity kinematics.

These findings are broadly consistent with previous studies showing good to excellent reliability of multi-camera MMC systems for many lower-extremity kinematic variables during gait and functional tasks (15, 18, 26, 31, 32).

### 4.1 Inter-system consistency of waveform and discrete metrics

Waveform analysis showed generally small inter-system differences across the gait cycle, with most parameters demonstrating limited RMSE magnitudes and only phase-specific significant differences. This agrees with previous MMC reliability studies, particularly using Theia3D, which reported low waveform variability and low full-curve error across many lower-extremity variables (8, 32, 33).

In several cases, statistically significant waveform differences occurred despite small RMSE values. This likely reflects the sensitivity of waveform-based statistical analyses to small but temporally consistent deviations (30). Therefore, SPM1D significance and RMSE were interpreted as complementary descriptors: SPM1D identifies temporally consistent inter-system differences, whereas RMSE reflects disagreement magnitude. Isolated significant SPM regions with low RMSE values were therefore not interpreted as clinically relevant disagreement.

The largest waveform deviations were observed for trunk flexion and selected transverse-plane variables. Trunk abduction and rotation showed low disagreement across groups, whereas trunk flexion consistently showed the highest sagittal-plane RMSE. Elevated trunk-flexion disagreement was present in all three groups, suggesting that a parameter-related source contributed to these errors. Nevertheless, the limited precision of the group comparisons means that an additional pathology-related contribution cannot be excluded. Previous studies have likewise reported lower reliability for transverse-plane variables (18, 32), and Yoma et al. (26) highlighted trunk kinematics as requiring further investigation.

A systematic offset was apparent in knee and hip transverse-plane kinematics, suggesting reduced robustness of longitudinal-axis rotation estimates. This is consistent with the known difficulty of estimating axial rotations in MMC, where visual landmarks provide less information for rotations around the segment long axis than for sagittal- or frontal-plane motion (3). Acquisition geometry may provide an additional source of disagreement. In a multicentre Theia3D study, Outerleys et al. (31) reported small centre-specific kinematic offsets and identified variation in camera setup, including differences in camera height imposed by spatial constraints, as a plausible contributor. Although the influence of camera placement could not be isolated from other site-related factors, the findings indicate that acquisition geometry may affect markerless kinematic estimates. In the present study, corresponding cameras differed by approximately 0.2–0.3 m in three-dimensional position, while both systems followed the same overall configuration and mounting strategy and neither was systematically positioned higher or lower. Small differences in camera geometry therefore cannot be excluded as a contributor to the observed inter-system disagreement, although their specific effect cannot be quantified from the present design.

These findings support a more cautious interpretation of transverse-plane hip and knee kinematics than of sagittal-plane lower-extremity variables. Similar limitations are also present in conventional marker-based gait analysis, where sagittal-plane variables generally show higher reliability and lower measurement error than hip and knee rotation in the transverse plane (28). The clinical relevance of the reduced consistency observed here therefore depends on the intended application. It may be of limited consequence for assessments primarily focused on sagittal-plane gait characteristics, but remains important when rotational abnormalities are of specific clinical interest or when three-dimensional kinematics are used as input for subsequent musculoskeletal modelling.

The discrete RoM results supported the waveform findings. Most parameters showed good or excellent ICC-based reliability, with small SEM and %SEM values. These results are consistent with previous Captury studies reporting moderate to excellent reliability across functional movement variables in healthy adults (14–17). The particularly favourable results for knee flexion agree with Lenhoff et al. (18), who also identified knee kinematics as among the most reliable Captury outputs. Bland–Altman analysis showed small systematic inter-system differences for most parameters, whereas trunk flexion showed larger bias and wider limits of agreement, confirming it as one of the least robust parameters. Proportional bias occurred only in a limited number of parameters and showed no consistent pattern across joints, planes, or groups.

Compared with the walking study by Lenhoff et al. (18), the present RoM reliability and measurement-error results were generally more favourable, while showing a similar pattern: knee flexion performed best, whereas transverse-plane variables were less robust. Differences in software product and version (CapturyLive v249 vs. CapturyStudio v2.8.5) and tracking mode may have contributed to the differences between studies. In particular, the multisampling mode used here performs repeated tracking passes to identify the best tracking result and may therefore affect tracking robustness compared with the processing mode used by Lenhoff et al. (18). However, the internal implementation of this procedure is not publicly documented. Software-related changes in MMC tracking performance have also been discussed for other systems (34). Software version and processing settings should therefore be considered part of the measurement protocol. For longitudinal studies, the exact software version and relevant processing settings should be reported and, where possible, kept consistent. If an update occurs during an ongoing study, retaining the original video recordings allows earlier data to be reprocessed using a common software version; alternatively, a bridging analysis in which identical recordings are processed with both versions can be used to quantify version-related differences before datasets are combined.

### 4.2 Influence of pathology on reliability

In the exploratory group analysis, the estimated differences did not show a systematic directional pattern of greater inter-system disagreement in the pathological groups. In particular, the healthy–pathological contrasts were not consistently larger than the contrast between the two pathological groups, which does not support a simple, uniform reduction in measurement consistency associated with the presence of pathology. Nevertheless, poorer reliability in specific joint–plane outcomes cannot be excluded, and the limited precision of the estimates prevents conclusions of equivalent reliability between groups.

The patient-reported outcome measures provide additional context for this comparison. Mean HOOS-12 and KOOS-12 summary scores were approximately 53 in the hip and knee groups, compared with substantially higher scores in the healthy controls. Scores around 50 correspond to moderate self-reported severity (35). Nevertheless, these patient-reported measures do not constitute radiographic disease grading, and the present data do not permit conclusions regarding whether measurement consistency varies with increasing disease severity.

An appendix analysis (see last Supplementary Figure) tested whether both systems detected similar between-group differences in subject-level RoM using system × group interaction models. No significant interaction effects were observed, providing no evidence that the systems differed in their sensitivity to group-related gait patterns. The clearest finding in both systems was reduced knee flexion RoM in the knee group compared with controls, which is biomechanically plausible and consistent with reduced sagittal-plane knee motion in degenerative knee pathology (36).

These findings are consistent with recent Theia3D results showing highly repeatable gait measurements in patients with knee osteoarthritis (33). Although the systems and statistical frameworks differ, both studies support the feasibility of MMC gait analysis in clinical populations with altered movement patterns.

### 4.3 Methodological and clinical implications

The use of two temporally aligned Captury systems enabled direct assessment of inter-system reliability and agreement under near-identical measurement conditions while avoiding between-session biological variability. This design isolates a lower bound on inter-system measurement disagreement; the implications of this scope restriction are discussed in the limitations below.

The combined use of waveform and discrete analyses provided complementary information. Waveform analysis identified temporally localised deviations across the gait cycle, whereas RoM summarised clinically interpretable movement characteristics. The inclusion of multiple gait cycles per participant further strengthened the analysis by reducing the influence of stride-to-stride variability. In older adults, Carvalho et al. (37) reported that averaging at least 7 strides per side was generally sufficient to obtain stable reliability estimates, with further improvement up to approximately 10 strides. The minimum of 15 gait cycles used here therefore supports the robustness of the subject-level estimates.

Participants wore their own everyday clothing, which may have introduced additional variability compared with standardised attire but improved ecological validity. The observed inter-system consistency indicates that comparable outputs can be achieved under the tested clothing conditions during simultaneous recordings. This is supported by previous Theia3D studies showing limited effects of clothing variation on inter-session kinematic variability (38, 39).

Clinically, reliable gait analysis is important for assessing impairments and monitoring functional outcomes after orthopaedic intervention. The present findings indicate that Captury can provide reproducible lower-extremity kinematics in older adults with altered gait patterns under the tested acquisition conditions. The consistent detection of reduced knee RoM in the knee group further suggests that the system can capture clinically meaningful between-group differences when present. However, this agreement between the two systems does not independently establish the validity or clinical sensitivity of the measurements.

### 4.4 Limitations

Several limitations should be considered. First, participants were recruited by convenience sampling during a predefined clinical recruitment period. No a priori precision- or power-based sample-size calculation was used. The group-specific reliability estimates should therefore be interpreted together with their confidence intervals, which reflect the uncertainty arising from the small number of independent participants. Although averaging at least 15 gait cycles per participant reduced within-participant sampling variability, the repeated gait cycles do not compensate for the limited number of participants when estimating population-level reliability or between-group effects. The secondary group comparisons were exploratory and were not designed to demonstrate equivalence or the absence of pathology-related differences. Consequently, the lack of FDR-adjusted group differences may reflect small underlying differences, limited statistical precision, or both. The findings support the conclusion that no consistent directional pattern was observed in this sample, but they cannot exclude clinically relevant increases in disagreement for individual outcomes. Future studies should prospectively define equivalence margins based on these results to rule out meaningfully greater inter-system disagreement in pathological populations.

Second, the participants represented a heterogeneous convenience sample of individuals with symptomatic hip or knee pathology. Patient-reported joint-related health was characterised using HOOS-12 and KOOS-12, but radiographic disease severity was not available and participants were not stratified according to symptom severity or functional limitation. Disease severity, pain, movement compensation, body composition, and functional limitation may affect markerless tracking, and the present study was not designed to estimate these effects individually. The reported estimates should therefore not be interpreted as representative of all severity levels of hip or knee disease. In addition, the healthy control group was recruited by convenience sampling from accompanying persons and was not age-matched to the pathological groups. The group comparison should therefore be interpreted as a pragmatic clinical comparison rather than a strictly age-matched case-control analysis. Future studies should include larger age-matched groups with sufficient age overlap to estimate age- and pathology-related effects separately and also include a broader spectrum of clinical impairment and examine whether inter-system reliability varies systematically with disease severity.

Third, some participants showed elevated influence diagnostics in the subject-level group models; however, excluding the most influential participants in a sensitivity analysis did not change the direction of any contrast or any FDR-adjusted conclusion. The full dataset was therefore retained for final inference.

Fourth, the study focused only on walking at a comfortable speed. Reliability may differ during tasks involving larger RoMs, faster movements, or more complex coordination, such as stair negotiation or sit-to-stand transitions. Future studies should examine whether the present findings generalise to other clinically relevant tasks.

Fifth, the study evaluated within-platform inter-system reliability between two identical Captury systems rather than validity or agreement with a marker-based reference system. The results therefore describe reproducibility within the Captury platform, not validity relative to conventional optical motion capture. This distinction is important because reliability does not imply interchangeability with marker-based data. MMC systems may be reliable for many outputs while still differing from ground truth depending on the task, joint, and movement plane (6, 7, 26, 40). Therefore, reliability testing is a necessary but not sufficient precondition for clinical use: a system that cannot reproduce its own output consistently cannot be meaningfully validated against an external reference, since any observed disagreement would be confounded by within-platform noise. The present design was deliberately scoped to establish this precondition in a population where it had not previously been tested; validity against marker-based or bi-planar reference systems in this cohort remains an essentially next step and should not be inferred from the present results.

Finally, the two Captury systems recorded simultaneously, which was a deliberate design choice to isolate inter-system measurement disagreement from other sources of variability that would otherwise confound its interpretation. This design necessarily excludes several sources of variability relevant to routine clinical use: session-to-session variability, differences in calibration performed at different time points, changes in participant clothing between visits, operator-dependent effects during setup and scaling, and variation in camera placement across installations or facilities. Because these factors were held constant by design, the reliability estimates reported here likely represent a best-case scenario, reflecting the platform’s intrinsic measurement consistency rather than the total reproducibility that clinicians would encounter across repeated clinical visits, where time-dependent biological variability further contributes to measurement error (41). Establishing the magnitude of these additional variance components is a necessary next step before the present reliability estimates can be generalised to everyday clinical practice. Regarding camera placement specifically, both systems in this study used broadly similar configurations, reflecting clinically intuitive best practice with lateral and anterior/posterior views (18); more divergent set-ups, such as tripod vs. truss-mounted placements (42), were not evaluated and may affect reliability differently.

## 5 Conclusion

In this convenience sample of adults with symptomatic hip or knee pathology and healthy controls, two simultaneously operated Captury systems showed generally small within-platform inter-system disagreement for most lower-limb gait kinematics. Point estimates of ICC-based reliability were predominantly good or excellent, and absolute measurement errors were small for most outcomes. Trunk flexion and selected transverse-plane rotations showed less consistent agreement and should be interpreted with greater caution.

Exploratory group comparisons did not reveal a consistent directional pattern of greater disagreement in the hip or knee groups. However, the small subgroup sizes and uncertainty of the estimated contrasts preclude conclusions that measurement consistency is equivalent between healthy and pathological populations.

The results therefore provide preliminary evidence of within-platform, inter-system consistency during walking. They do not establish validity against a reference system, between-session reproducibility or generalisability to all older clinical populations.

## Data Availability

The raw data supporting the conclusions of this article will be made available by the authors, without undue reservation.
