# Supplementary material for: Inter-system reliability of markerless motion capture in older hip and knee patients during walking
Source: Front Med Technol. 2026 Oct 15;8:1940851. doi: 10.3389/fmedt.2026.1940851 (PMC13716743; doi:10.3389/fmedt.2026.1940851)
Supplement: Supplementary file 1 [file Datasheet1.pdf]

frontiers

Supplementary Material

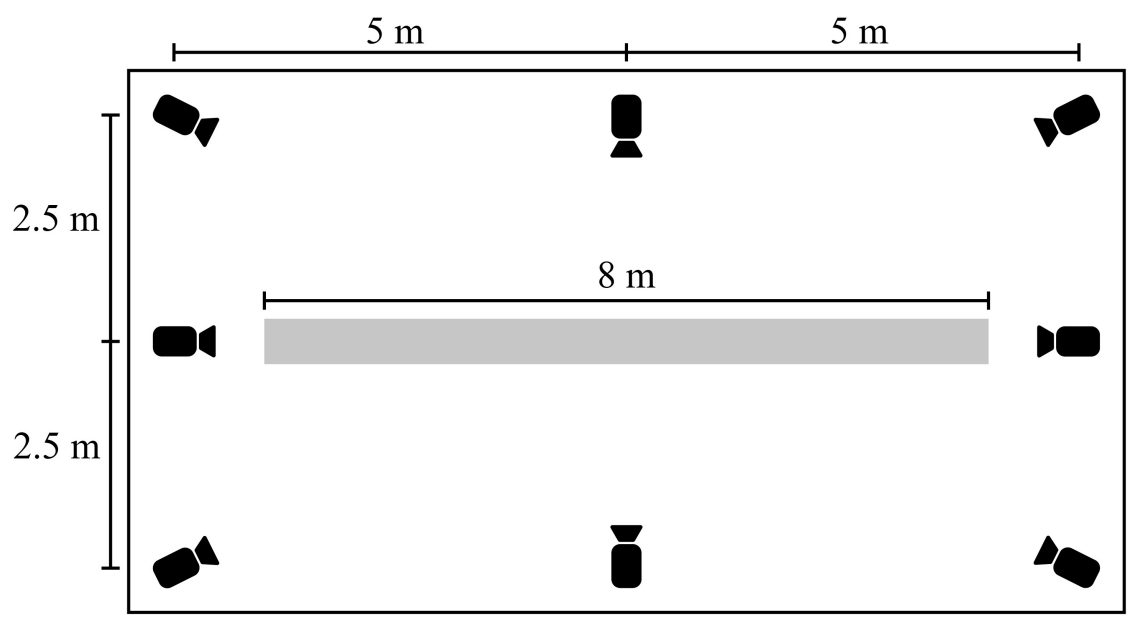

Figure S1. Camera setup schematic for one markerless motion capture (MMC) system. Each system comprised eight cameras distributed around an approximately 10 m $\times$ 5 m rectangular perimeter surrounding the 8-m walkway. Six cameras were wall-mounted and the two opposing lateral cameras were tripod-mounted. Corresponding camera positions between the two systems differed by approximately 0.2 – 0.3 m in three-dimensional space to represent two practically equivalent but not geometrically identical set-ups. Orientations were subsequently adjusted to ensure that all cameras were directed towards the centre of the walkway. Camera heights ranged from 1.5 to 2.5 m.

Table S1. Subject-level group differences in inter-system disagreement for discrete range of motion (RoM). For each joint-plane combination, group contrasts are reported as the first group minus the second group named in the contrast. Mean_1 [SD] and Mean_2 [SD] denote the group-specific subject-level disagreement values for the first and second group, respectively. Difference [95% CI] denotes the estimated between-group difference with its 95% confidence interval. Raw and Benjamini–Hochberg false discovery rate (FDR)-adjusted p-values are reported as p_{raw} and p_{FDR}, respectively; inferential interpretation was based on p_{FDR}. Discrete disagreement was defined as the subject-level mean absolute between-system difference in RoM. All analyses were based on one three-group linear model per outcome with HC3 robust standard errors and post hoc pairwise contrasts.

Joint	Plane	Contrast	Mean_1 [SD] / °	Mean_2 [SD] / °	Difference [95% CI] / °	p_{raw}	p_{FDR}
Trunk	Flexion	healthy:hip	3.69 [3.10]	3.34 [1.23]	0.35 [-1.52, 2.22]	0.71	0.83
		healthy:knee	3.69 [3.10]	5.38 [4.09]	-1.69 [-4.59, 1.22]	0.25	0.76
		hip:knee	3.34 [1.23]	5.38 [4.09]	**-2.04** [-4.44, 0.36]	0.10	0.49
	Abduction	healthy:hip	1.59 [0.70]	1.75 [0.78]	-0.16 [-0.73, 0.40]	0.57	0.82
		healthy:knee	1.59 [0.70]	2.12 [0.82]	-0.53 [-1.14, 0.08]	0.09	0.49
		hip:knee	1.75 [0.78]	2.12 [0.82]	-0.36 [-0.98, 0.25]	0.25	0.76
	Rotation	healthy:hip	0.68 [0.31]	0.63 [0.19]	0.05 [-0.16, 0.25]	0.65	0.82
		healthy:knee	0.68 [0.31]	0.88 [0.57]	-0.20 [-0.56, 0.17]	0.29	0.81
		hip:knee	0.63 [0.19]	0.88 [0.57]	-0.24 [-0.58, 0.09]	0.16	0.63
Hip	Flexion	healthy:hip	2.89 [1.81]	2.50 [0.59]	0.39 [-0.68, 1.46]	0.47	0.81
		healthy:knee	2.89 [1.81]	3.40 [1.59]	-0.51 [-1.88, 0.85]	0.46	0.81
		hip:knee	2.50 [0.59]	3.40 [1.59]	-0.90 [-1.85, 0.04]	0.06	0.44
	Abduction	healthy:hip	1.31 [0.51]	1.24 [0.37]	0.08 [-0.27, 0.43]	0.66	0.82
		healthy:knee	1.31 [0.51]	1.43 [0.37]	-0.12 [-0.48, 0.24]	0.51	0.81
		hip:knee	1.24 [0.37]	1.43 [0.37]	-0.20 [-0.48, 0.09]	0.17	0.63
	Rotation	healthy:hip	4.84 [1.91]	4.62 [1.25]	0.21 [-1.05, 1.48]	0.74	0.83
		healthy:knee	4.84 [1.91]	4.32 [0.91]	0.51 [-0.69, 1.71]	0.40	0.81
		hip:knee	4.62 [1.25]	4.32 [0.91]	0.30 [-0.53, 1.13]	0.48	0.81
Knee	Flexion	healthy:hip	1.25 [0.50]	1.25 [0.39]	-0.00 [-0.35, 0.35]	1.00	1.00
		healthy:knee	1.25 [0.50]	1.67 [0.51]	-0.42 [-0.83, -0.02]	**0.04**	0.44
		hip:knee	1.25 [0.39]	1.67 [0.51]	-0.42 [-0.78, -0.07]	**0.02**	0.35
	Abduction	healthy:hip	1.08 [0.36]	1.15 [0.25]	-0.06 [-0.31, 0.18]	0.60	0.82
		healthy:knee	1.08 [0.36]	1.16 [0.27]	-0.08 [-0.34, 0.18]	0.54	0.81
		hip:knee	1.15 [0.25]	1.16 [0.27]	-0.02 [-0.22, 0.18]	0.88	0.93
	Rotation	healthy:hip	3.05 [1.19]	3.06 [1.20]	-0.02 [-0.93, 0.90]	0.97	1.00
		healthy:knee	3.05 [1.19]	3.33 [1.01]	-0.29 [-1.17, 0.60]	0.53	0.81
		hip:knee	3.06 [1.20]	3.33 [1.01]	-0.27 [-1.12, 0.58]	0.53	0.81
Ankle	Flexion	healthy:hip	2.31 [0.94]	2.09 [0.61]	0.23 [-0.39, 0.85]	0.48	0.81
		healthy:knee	2.31 [0.94]	2.46 [0.67]	-0.15 [-0.80, 0.51]	0.66	0.82
		hip:knee	2.09 [0.61]	2.46 [0.67]	-0.37 [-0.86, 0.12]	0.14	0.63
	Abduction	healthy:hip	0.01 [0.01]	0.01 [0.00]	0.00 [-0.00, 0.00]	0.69	0.83
		healthy:knee	0.01 [0.01]	0.01 [0.00]	0.00 [-0.00, 0.00]	0.52	0.81
		hip:knee	0.01 [0.00]	0.01 [0.00]	0.00 [-0.00, 0.00]	0.83	0.91
	Rotation	healthy:hip	2.58 [0.80]	3.15 [0.65]	-0.56 [-1.13, 0.01]	0.05	0.44
		healthy:knee	2.58 [0.80]	3.42 [0.95]	-0.84 [-1.54, -0.14]	**0.02**	0.35
		hip:knee	3.15 [0.65]	3.42 [0.95]	-0.28 [-0.91, 0.36]	0.39	0.81

Table S2. Subject-level group differences in inter-system waveform disagreement quantified by root-mean-square error (RMSE). For each joint-plane combination, group contrasts are reported as the first group minus the second group named in the contrast. $Mean_1$ [SD] and $Mean_2$ [SD] denote the group-specific subject-level disagreement values for the first and second group, respectively. Difference [95% CI] denotes the estimated between-group difference with its 95% confidence interval. Raw and Benjamini–Hochberg false discovery rate (FDR)-adjusted p-values are reported as p_{raw} and p_{FDR}, respectively; inferential interpretation was based on p_{FDR}. Waveform disagreement was defined as the between-system subject-level RMSE across the normalised gait cycle. All analyses were based on one three-group linear model per outcome with HC3 robust standard errors and post hoc pairwise contrasts.

Joint	Plane	Contrast	$Mean_1$ [SD] / °	$Mean_2$ [SD] / °	Difference [95% CI] / °	p_{raw}	p_{FDR}
Trunk	Flexion	healthy:hip	5.52 [3.83]	6.34 [3.62]	-0.82 [-3.70, 2.06]	0.58	0.77
		healthy:knee	5.52 [3.83]	8.98 [4.23]	**-3.46** [-6.69, -0.24]	**0.04**	0.20
		hip:knee	6.34 [3.62]	8.98 [4.23]	**-2.64** [-5.69, 0.41]	0.09	0.20
	Abduction	healthy:hip	1.53 [0.61]	1.59 [0.46]	-0.06 [-0.48, 0.36]	0.78	0.83
		healthy:knee	1.53 [0.61]	2.10 [0.95]	-0.58 [-1.21, 0.06]	0.08	0.20
		hip:knee	1.59 [0.46]	2.10 [0.95]	-0.52 [-1.11, 0.07]	0.09	0.20
	Rotation	healthy:hip	0.64 [0.16]	0.63 [0.16]	0.00 [-0.12, 0.13]	0.94	0.94
		healthy:knee	0.64 [0.16]	0.93 [0.48]	-0.29 [-0.58, -0.01]	**0.04**	0.20
		hip:knee	0.63 [0.16]	0.93 [0.48]	-0.30 [-0.58, -0.01]	**0.04**	0.20
Hip	Flexion	healthy:hip	3.97 [2.55]	4.40 [2.28]	-0.42 [-2.30, 1.45]	0.66	0.79
		healthy:knee	3.97 [2.55]	5.89 [2.62]	-1.92 [-3.98, 0.15]	0.07	0.20
		hip:knee	4.40 [2.28]	5.89 [2.62]	-1.49 [-3.39, 0.41]	0.12	0.26
	Abduction	healthy:hip	1.14 [0.28]	1.38 [0.35]	-0.24 [-0.48, 0.01]	0.06	0.20
		healthy:knee	1.14 [0.28]	1.59 [0.45]	-0.45 [-0.75, -0.15]	**¡0.01**	0.10
		hip:knee	1.38 [0.35]	1.59 [0.45]	-0.21 [-0.53, 0.10]	0.18	0.34
	Rotation	healthy:hip	10.69 [4.15]	8.18 [3.15]	**2.51** [-0.36, 5.39]	0.09	0.20
		healthy:knee	10.69 [4.15]	10.81 [3.36]	-0.11 [-3.14, 2.91]	0.94	0.94
		hip:knee	8.18 [3.15]	10.81 [3.36]	**-2.63** [-5.14, -0.11]	**0.04**	0.20
Knee	Flexion	healthy:hip	1.65 [0.54]	1.60 [0.30]	0.05 [-0.29, 0.39]	0.76	0.83
		healthy:knee	1.65 [0.54]	2.02 [0.45]	-0.37 [-0.76, 0.03]	0.07	0.20
		hip:knee	1.60 [0.30]	2.02 [0.45]	-0.42 [-0.72, -0.12]	**¡0.01**	0.10
	Abduction	healthy:hip	1.01 [0.29]	1.09 [0.35]	-0.08 [-0.33, 0.17]	0.53	0.73
		healthy:knee	1.01 [0.29]	1.14 [0.22]	-0.13 [-0.34, 0.08]	0.22	0.39
		hip:knee	1.09 [0.35]	1.14 [0.22]	-0.05 [-0.27, 0.17]	0.66	0.79
	Rotation	healthy:hip	8.88 [3.23]	6.61 [2.49]	**2.27** [0.03, 4.52]	**0.05**	0.20
		healthy:knee	8.88 [3.23]	8.34 [3.27]	0.54 [-2.06, 3.14]	0.68	0.79
		hip:knee	6.61 [2.49]	8.34 [3.27]	-1.73 [-3.99, 0.53]	0.13	0.27
Ankle	Flexion	healthy:hip	3.03 [0.86]	2.71 [0.80]	0.32 [-0.33, 0.96]	0.34	0.53
		healthy:knee	3.03 [0.86]	3.59 [1.41]	-0.56 [-1.49, 0.38]	0.24	0.41
		hip:knee	2.71 [0.80]	3.59 [1.41]	-0.87 [-1.78, 0.03]	0.06	0.20
	Abduction	healthy:hip	0.01 [0.00]	0.01 [0.00]	0.00 [-0.00, 0.00]	0.73	0.82
		healthy:knee	0.01 [0.00]	0.01 [0.00]	-0.00 [-0.00, 0.00]	0.66	0.79
		hip:knee	0.01 [0.00]	0.01 [0.00]	-0.00 [-0.00, 0.00]	0.39	0.56
	Rotation	healthy:hip	3.34 [0.76]	3.86 [1.47]	-0.52 [-1.40, 0.36]	0.25	0.41
		healthy:knee	3.34 [0.76]	4.39 [1.62]	-1.05 [-2.06, -0.03]	**0.04**	0.20
		hip:knee	3.86 [1.47]	4.39 [1.62]	-0.53 [-1.72, 0.67]	0.39	0.56

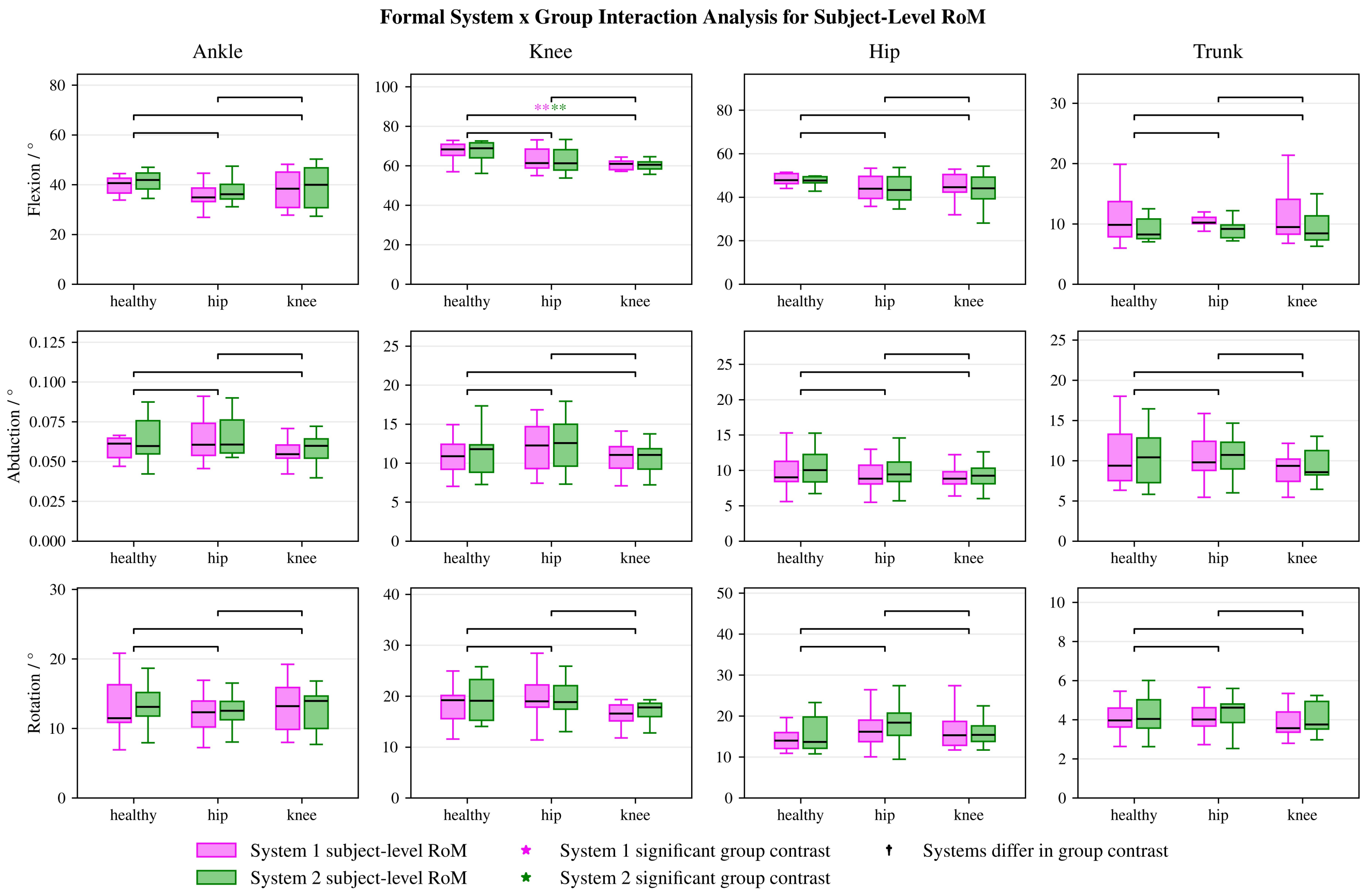

Figure S2. Formal system × group interaction analysis for subject-level range of motion (RoM). For each joint-plane outcome, box plots show subject-level RoM values from the two synchronised markerless motion-capture systems for the healthy, hip pathology, and knee pathology groups. Magenta and green symbols denote the two systems. Asterisks indicate significant pairwise group contrasts within a given system, whereas daggers indicate that the corresponding group contrast differed significantly between systems (system × group interaction). Significance was based on Benjamini–Hochberg false discovery rate-adjusted p-values.
